# Counteracting Roles of AMP Deaminase and AMP Kinase in the Development of Fatty Liver

**DOI:** 10.1371/journal.pone.0048801

**Published:** 2012-11-09

**Authors:** Miguel A. Lanaspa, Christina Cicerchi, Gabriela Garcia, Nanxing Li, Carlos A. Roncal-Jimenez, Christopher J. Rivard, Brandi Hunter, Ana Andrés-Hernando, Takuji Ishimoto, Laura G. Sánchez-Lozada, Jeffrey Thomas, Robert S. Hodges, Colin T. Mant, Richard J. Johnson

**Affiliations:** 1 Division of Renal Diseases and Hypertension, University of Colorado-Denver, Aurora, Colorado, United States of America; 2 Department of Biochemistry and Molecular Genetics, University of Colorado-Denver, Aurora, Colorado, United States of America; University of Las Palmas de Gran Canaria, Spain

## Abstract

Fatty liver (hepatic steatosis) is associated with nucleotide turnover, loss of ATP and generation of adenosine monophosphate (AMP). It is well known that in fatty liver, activity of the AMP-activated kinase (AMPK) is reduced and that its stimulation can prevent hepatic steatosis by both enhancing fat oxidation and reducing lipogenesis. Here we show that another AMP dependent enzyme, AMPD2, has opposing effects on fatty acid oxidation when compared to AMPK. In human hepatocytres, AMPD2 activation –either by overexpression or by lowering intracellular phosphate levels with fructose- is associated with a significant reduction in AMPK activity. Likewise, silencing of AMPK spontaneously increases AMPD activity, demonstrating that these enzymes counter-regulate each other. Furthermore, we show that a downstream product of AMP metabolism through AMPD2, uric acid, can inhibit AMPK activity in human hepatocytes. Finally, we show that fructose-induced fat accumulation in hepatocytes is due to a dominant stimulation of AMPD2 despite stimulating AMPK. In this regard, AMPD2-deficient hepatocytes demonstrate a further activation of AMPK after fructose exposure in association with increased fatty acid oxidation, and conversely silencing AMPK enhances AMPD-dependent fat accumulation. In vivo, we show that sucrose fed rats also develop fatty liver that is blocked by metformin in association with both a reduction in AMPD activity and an increase in AMPK activity. In summary, AMPD and AMPK are both important in hepatic fat accumulation and counter-regulate each other. We present the novel finding that uric acid inhibits AMPK kinase activity in fructose-fed hepatocytes thus providing new insights into the pathogenesis of fatty liver.

## Introduction

There has been a progressive increase in obesity, type 2 diabetes, dyslipidemia and fatty liver throughout the world [1]. In particular, there has been an increasing frequency of nonalcoholic hepatic steatosis in subjects with obesity or insulin resistance that may progress to chronic liver disease [2], [3].

Today, it is accepted that the inhibition of the energy sensor protein AMPK (AMP activated kinase) is an important step in the development of fatty liver [4], [5], [6]. AMPK activity is reduced in fatty liver and its inhibition is associated with fat accumulation. In this regard, AMPK modulates hepatic lipogenesis by multiple mechanisms including the phosphorylation (and inactivation) of transcription factors including SREBP-1c and -2 [7], [8], [9] and ChREBP [10] resulting in the inhibition of the transcription of lipogenic target genes FAS (fatty acid synthase), ACC1 (acetyl-CoA carboxylase) and SCD1 (stearoyl-CoA desaturase) [7], [8], [9]. AMPK can also directly inactivate lipogenesis by phosphorylation of ACC1 at Ser79 [11], [12]. On the other hand, AMPK stimulates fat oxidation both by inhibiting ACC1 activity and shifting malonyl-CoA to the mitochondria [11] and transcriptionally through the activation of PPARα and its downstream target genes [13], [14]. AMPK agonists have also been shown to have protective effects in fatty liver. While a reduction in AMPK activity is important in the development of hepatic steatosis, less is known about the mechanisms whereby AMPK activity is blocked in this condition.

In this manuscript, we characterize the activity of another AMP dependent protein, AMPD2 (AMP deaminase 2 [15],[16]) in an in vitro and in vivo model of hepatic steatosis, fructose-induced fatty liver [17], [18]. Since both AMPK and AMPD2 require AMP as a substrate, we studied whether the activation of AMPD2 in the settings where fructose is present may have countering effects to AMPK. Activation of AMPD2 blocks fat oxidation which is mediated by both inhibiting AMPK activity and by generating uric acid from AMP. In contrast, activating AMPK blocks AMPD2 activity in the liver in addition to directly stimulating fat oxidation. We also show that fructose preferentially engages AMPD2 over AMPK and causes fat accumulation. We suggest that AMP, which is a vestigial molecule carried over from the RNA world [19], sits on a fulcrum in which it may engage AMPD2 to cause fat accumulation or AMPK to stimulate fat oxidation. Hence, we suggest that the activation of AMPK or AMPD represent a metabolic “switch” that stimulates fat oxidation or fat accumulation, respectively.

## Results

### Activation of AMPK Stimulates Fat Oxidation in HepG2 Cells

**Figure 1:**
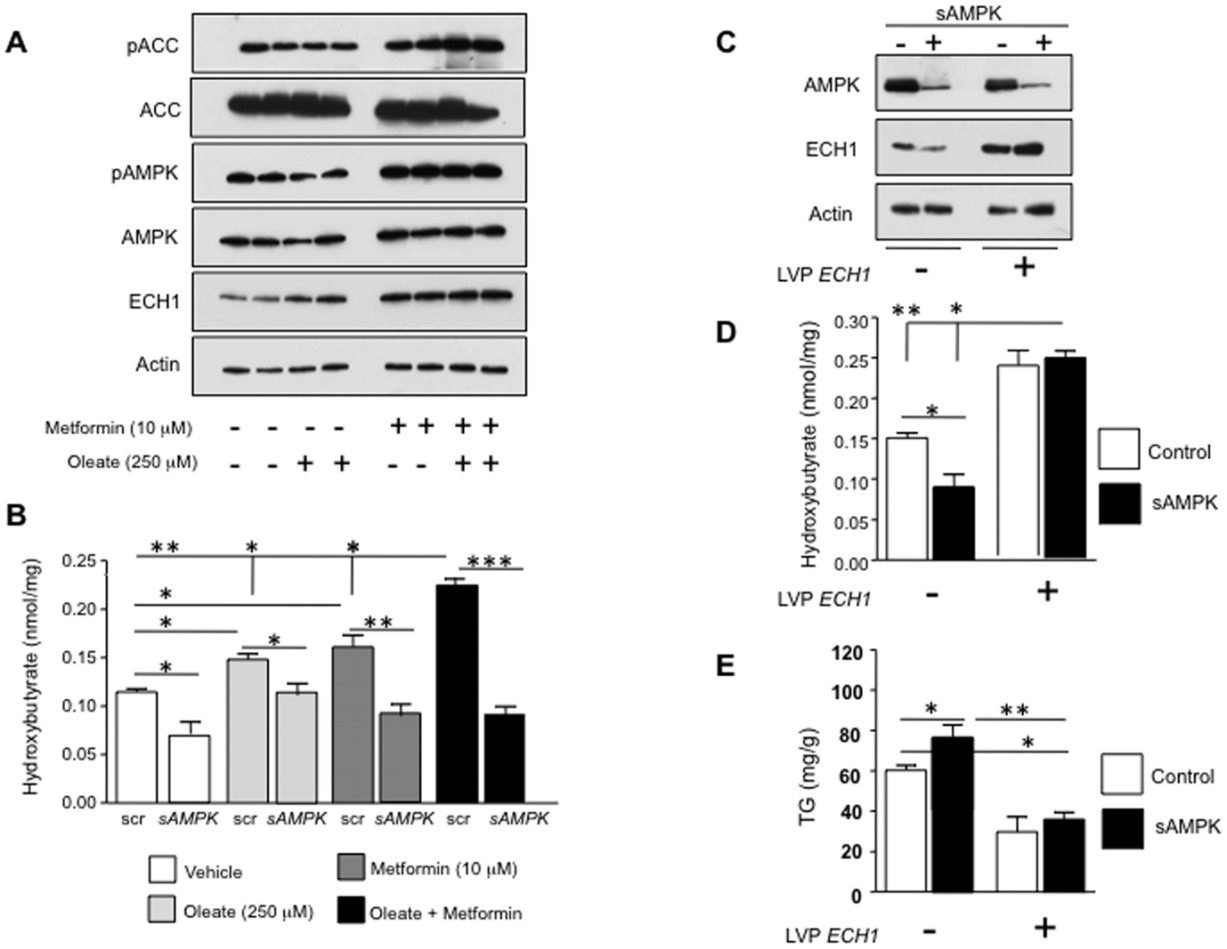
Activation of AMPK stimulates fat oxidation in HepG2 cells. A) Stimulation of AMPK activity by metformin (10 µM) significantly increases the phosphorylation of AMPK at Thr172, the phosphorylation of ACC at ser79 as well as the expression of the fat oxidation-related protein ECH1 (Enoyl CoA-hydratase) B) Metformin up-regulates the accumulation of β-hydroxybutyrate -a marker of fat oxidation activity- in cells exposed to oleic acid (250 µM, 72 hour). Hydroxybutyrate accumulation, ACC phosphorylation and ECH1 up-regulation by metformin does not occur in AMPK-deficient cells. *p<0.05, **p<0.01, ***p<0.001 C) Representative western blot showing over-expression of ECH1 in HepG2 cells (lanes 3 and 4) in control cells (lanes 1 and 3) and AMPK deficient cells (lanes 2 and 4). ECH1 overexpression restores the loss of fat oxidation observed in AMPK deficient cells and reduces fat accumulation as determined by a significant up-regulation of β-hydroxybuyrate levels (D) with paralleled decrease in intracellular triglyceride levels (E) *p<0.05, **p<0.01.

Metformin is widely considered an activator of AMPK [20]. Consistent with this finding, metformin (10 µM) increased the phosphorylation of threonine 172 (pAMPK), which is considered key in the activation of AMPK [21] (Fig. 1A and Fig. S1A). Consistent with the activation of AMPK, phosphorylation of ACC at serine 79, a well-established AMPK target gene [12], is increased in hepatocytes upon incubation with metformin. Of interest, metformin also increased the expression of enoyl CoA hydratase-1 (ECH1), an enzyme in β-fatty acid oxidation [22] resulting in increased levels of the ketone β-hydroxybutyrate [23]. These effects were amplified in the presence of the fatty acid substrate, oleate (250 µM) (Fig. 1B).

To determine whether the stimulation of fat oxidation by metformin is dependent on AMPK, we developed stable cell lines deficient in AMPK expression and observed that metformin was ineffective in increasing both ECH1 expression, the phosphorylation of ACC at serine 79 and the levels of intracellular β-hydroxybutyrate in AMPK-deficient cells (Fig. 1B and figure S1B-C) indicating that AMPK controls fat oxidation by regulating ECH1 expression. We also investigated the role of ECH1 in AMPK-dependent fat oxidation by generating cell lines where ECH1 was stably over-expressed (Fig. 1C, top). Overexpression of ECH1 in HepG2 cells restored the loss of fat oxidation in AMPK-deficient cells (Fig. 1C, middle) resulting in decreased triglyceride accumulation in response to oleate (Fig. 1C, bottom).

**Figure 2:**
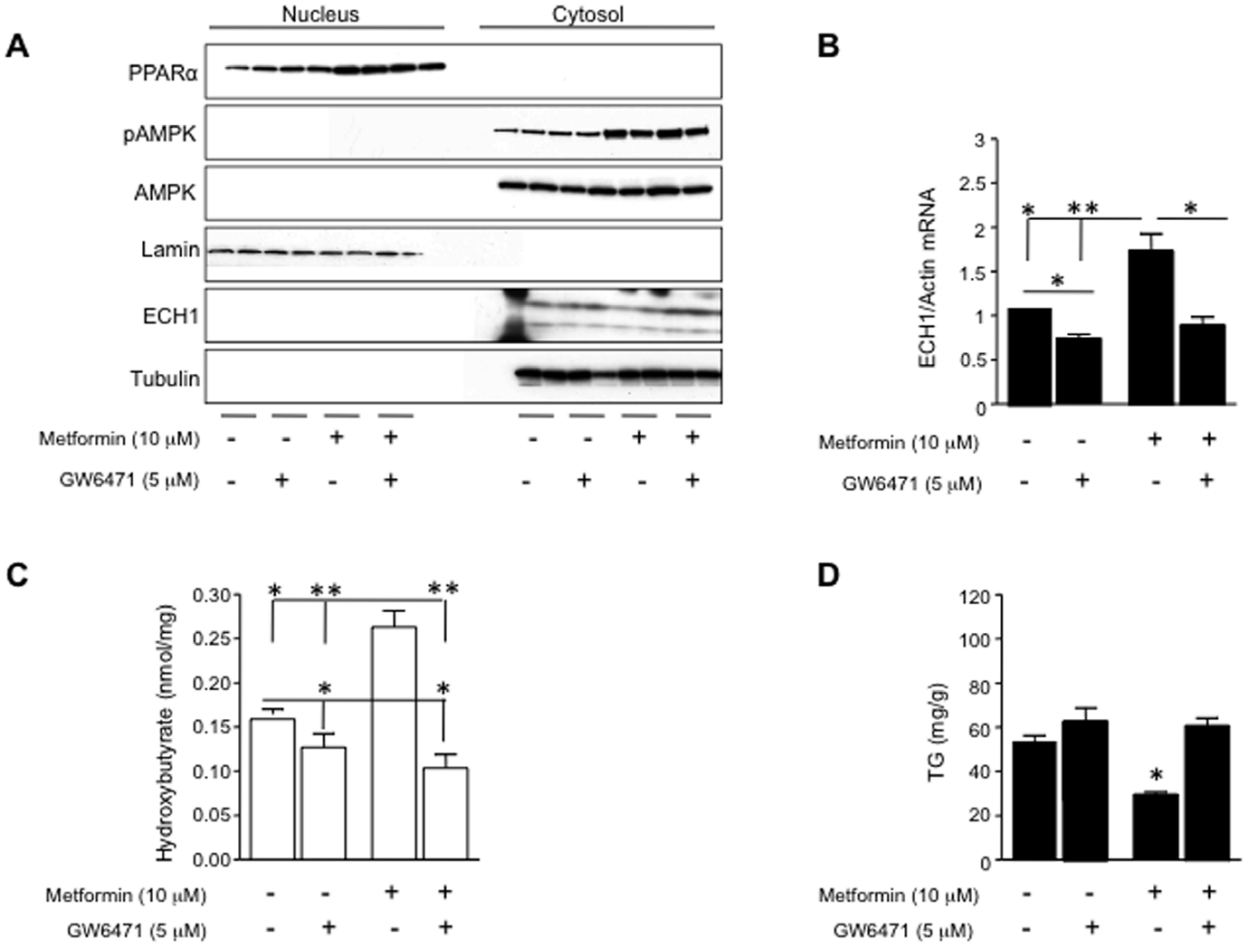
Metformin-induced up-regulation of ECH1 is mediated by PPARα. A) Representative western blot of nuclear and cytosolic extracts from hepatocytes exposed to metformin (10 µM) and the PPARα inhibitor, GW6471 (5 µM). As shown, GW6471 reduces metformin-induced ECH1 up-regulation in the cytoplasmic fraction but do not affet either PPARα nuclear expression or AMPK phosphorylation. B) ECH1 mRNA levels in hepatocytes exposed to metformin (10 µM) and the PPARα inhibitor, GW6471 (5 µM) C) Inhibition of PPARα activity with the antagonist GW6471 reduces metformin-induced fat oxidation. D) Inhibition of PPARα activity with the antagonist GW6471 reduces metformin-induced trigyceride accumulation. *p<0.05, **p<0.01.

ECH1 is a target gene of the transcription factor, PPARα [24], [25]. To determine whether the mechanism whereby AMPK stimulates ECH1 expression could be mediated by PPARα, we exposed HepG2 cells to the specific PPARα antagonist GW6471 (5 µM) and analyzed ECH1 expression and fat oxidation in the presence of oleate, and metformin. As shown in Fig. 2A, GW6471 reduced metformin-induced ECH1 up-regulation in the cytosol but did not affect either AMPK phosphorylation or PPARα nuclear expression. Inhibition of PPARα with GW6471 also blocked the increase in both metformin-induced ECH1 mRNA levels (Fig. 2B) and β-hydroxybutyrate levels (Fig. 2C) resulting in reduced triglyceride accumulation in response to oleate (Fig. 2D). This suggests GW6471 blocks metformin-induced fat oxidation by inhibiting the transcriptional activity of PPARα rather than inhibiting its nuclear localization.

### AMPD2 is the Main Isoform of AMPD in HepG2 Cells where it Modulates the Activity of AMPK

**Figure 3:**
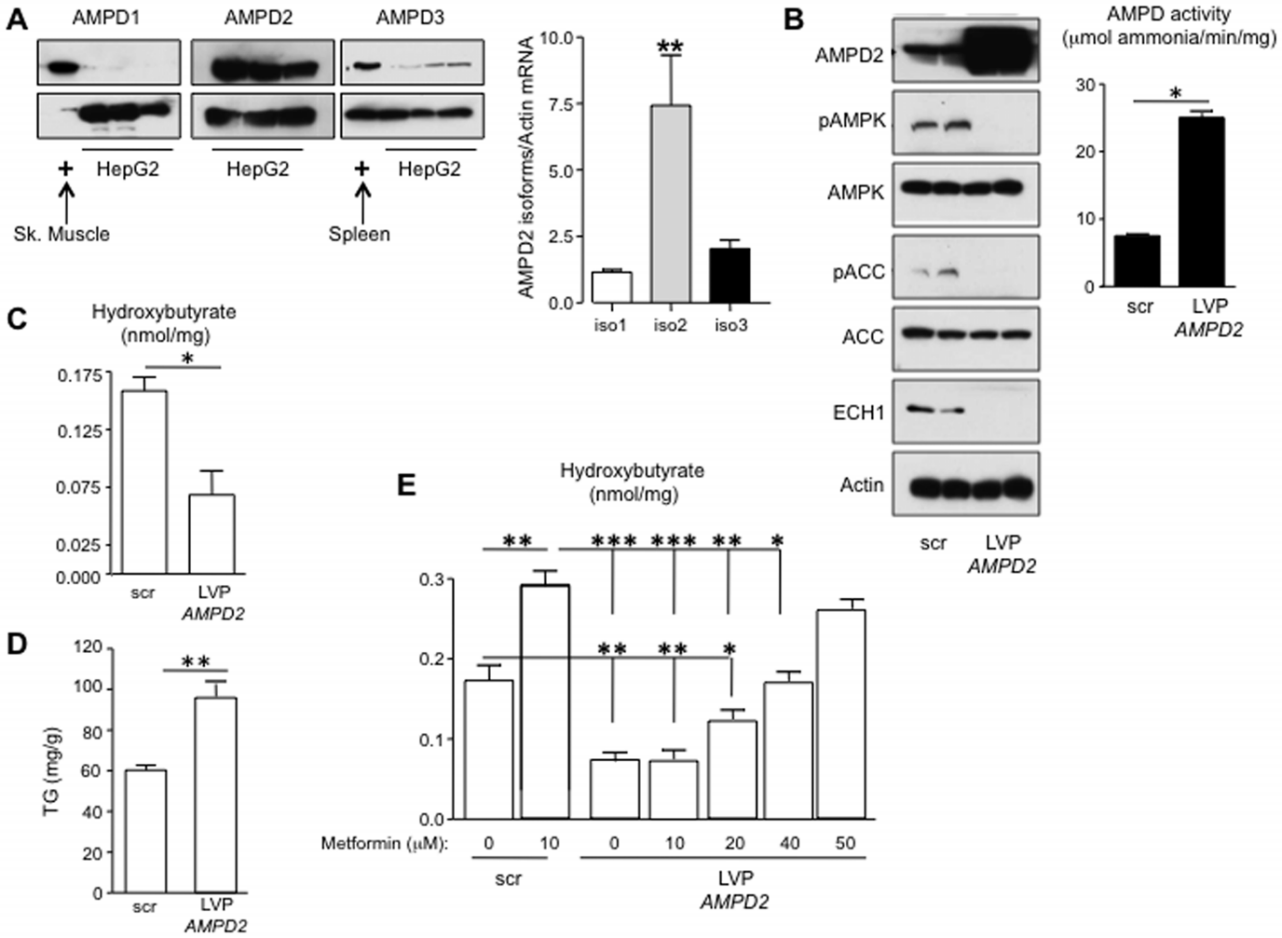
Metformin regulates AMPD2 activity in human hepatocytes. A) AMPD2 is the main isoform in HepG2 cells. The expression of AMPD1 and AMPD3 isoforms is minimal compred to AMPD2. Skeletal muscle and spleen are positive controls for AMPD1 and AMPD3 expression, respectively. Quantitative PCR analysis (bottom) demonstrates that isoform 2 is the predominant isoform of AMPD2. **p<0.01 versus isoforms 1 and 3. B) Over-expression of AMPD2 down-regulates the activation of AMPK. Transduction of HepG2 cells with lentiviral particles codifying for the isoform 2 of AMPD2 results in significantly higher levels of AMPD2 protein expression as well as AMPD activity. This is paralleled with reduced levels Thr172 pAMPK expression as well as of their target genes ECH1 and Ser79 pACC. *p<0.05 C–D) Reduction in ECH1 expression in over-expressing AMPD2 hepatocytes is accompanied with lower intracellular β-hydroxybutyrate and higher TG levels. *p<0.05, **p<0.01 E) Over-expression of AMPD2 impairs metformin-induced fat oxidation. Metformin 10 µM significantly increased β- hydroxybutyrate levels in cells transducted with scramble RNA. In contrast, no significant change was observed with 10 µM metformin in cells overexpressing AMPD2. Metformin 50 µM significantly increased β- hydroxybutyrate to levels observed in scramble transducted cells *p<0.05, **p<0.01, ***p<0.001.

After determining that AMPK activation triggers fat oxidation in HepG2 cells, we characterized the activity another AMP-dependent enzyme, AMP Deaminase (AMPD). HepG2 cells primarily express the AMPD2 isoform which is the principal AMPD isoform expressed in the liver [15], with lower expression of AMPD3 and little or no expression of AMPD1 (Fig. 3A, top). Quantitative PCR showed that of the three different splice variants of AMPD2, AMPD2 isoform 2 is the main variant in this cell line (Fig. 3A, bottom). To determine whether AMPD2 modulates AMPK activity, we overexpressed AMPD2 isoform 2 (AMPD2) in HepG2 cells. Overexpression of AMPD2 led to a marked increase in AMPD activity and a spontaneous reduction of active AMPK (pAMPK) levels (Fig. 3B). This was associated with a significant decrease in ECH1 levels and phosphorylated acetyl carboxylase at serine 79 (pACC), an established target protein of pAMPK (Fig. 3B). The increased expression of AMPD2 resulted in a significant reduction in β-hydroxybutyrate levels with a parallel increase in TG levels in response to oleate (Fig. 3C–D). These studies show that increasing AMPD activity inhibits AMPK activation and β-fatty acid oxidation.

We next examined whether AMPK stimulation by metformin could counteract the overexpression of AMPD2. In contrast to normal cells where 10 µM metformin significantly increased β-hydroxybutyrate levels after 72 hours of exposure (Fig. 3E, left), the same concentration of metformin failed to increase β-hydroxybutyrate levels in AMPD2 overexpressing cells. Indeed, five times the dose of metformin (50 µM) was required to generate similar levels of β-hydroxybutyrate (Fig. 3E, right). This suggests that increased AMPD2 activity down-regulates AMPK activity in liver cells resulting in the blockade of fat oxidation.

**Figure 4:**
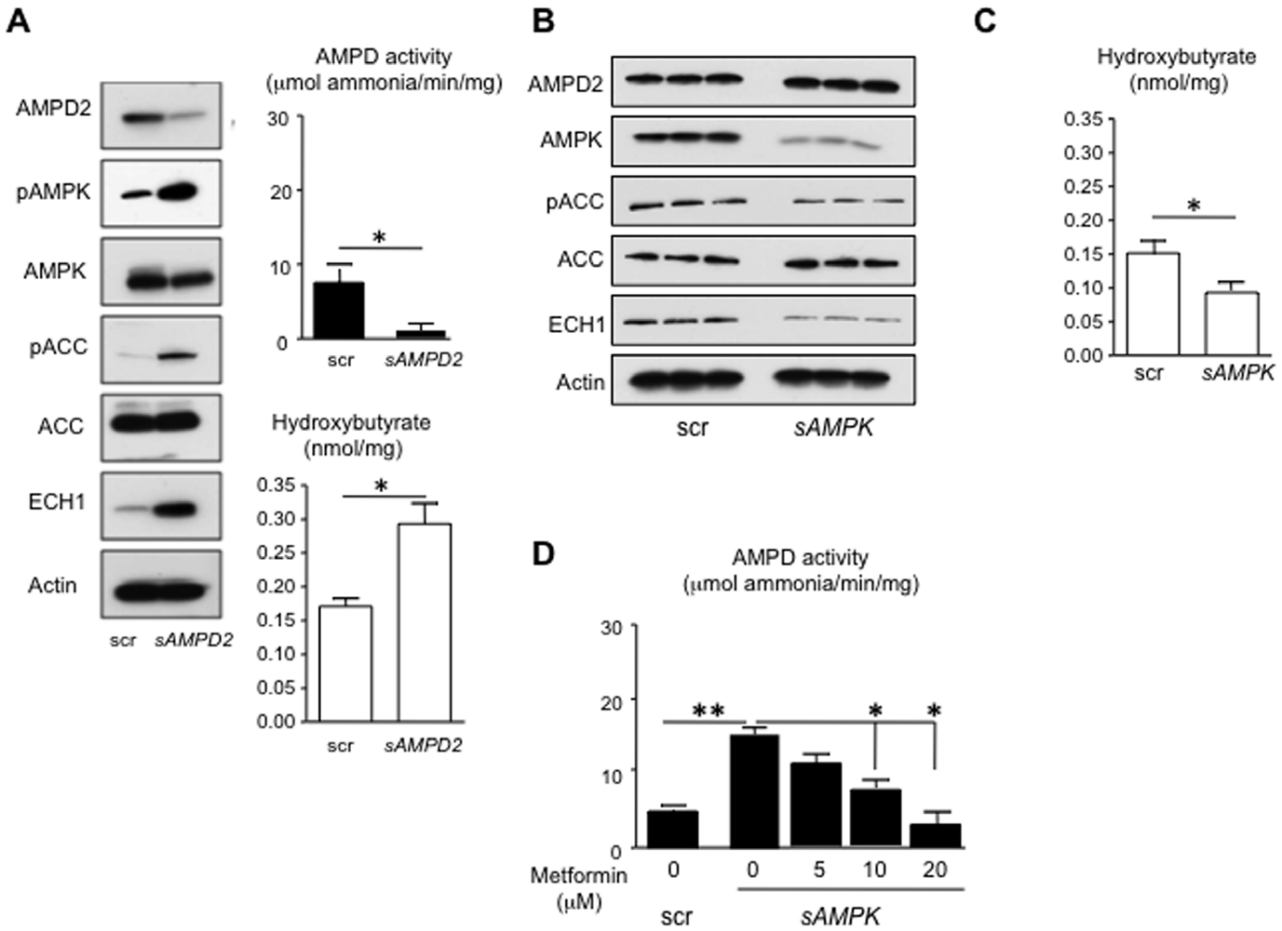
AMPK activation in AMPD2-deficient cells. A) Silencing AMPD2 expression in human hepatocytes spontaneously up-regulates the activation of AMPK. Transduction of HepG2 cells with lentiviral particles codifying for a specific silencer for AMPD2 results in significantly lower levels of AMPD activity. This is paralleled with increaseded levels Thr172 pAMPK expression as well as of their target genes ECH1 and Ser79 pACC. Up-regulation of ECH1 is accompanied with higher intracellular β-hydroxybutyrate levels. *p<0.05 B) Blockade of AMPK expression activates AMPD2. Stable silencing of AMPK is associated with significant down-regulation of ECH1 and phosphorylation of ACC at Ser79. In contrast, no change in AMPD2 expression is observed. C) Silencing AMPK expression in human hepatocytes spontaneously decreases the levels of intracellular β-hydroxybutyrate D) Metformin blocks AMPD activation in AMPK-deficient cells in a dose-dependent manner. *p<0.05, **p<0.01.

We also tested the effect of silencing AMPD2 to evaluate its effect on AMPK activity. HepG2 cells silenced for AMPD2 showed a significant reduction in AMPD activity (Fig. 4A) with stimulation of pAMPK, increased pACC and ECH1 by western blot analysis in association with increased β-hydroxybutyrate levels (Fig. 4A). These studies show that AMPD2 activity regulates AMPK activity.

### Blockade of AMPK Expression Activates AMPD2 and Induces Fat Oxidation in HepG2 Cells

The observation that AMPD regulates AMPK does not exclude the reverse possibility that AMPK may also regulate AMPD. To evaluate this possibility, we stably silenced isoforms 1 and 2 of the α-subunit of AMPK in HepG2 cells. As shown in Fig. 4B, blockade of AMPK expression in HepG2 cells resulted in a significant reduction in pACC and ECH1 expression confirming efficient AMPK silencing. The reduction in ECH1 expression in AMPK deficient cells resulted in significantly lower basal β-hydroxybutyrate levels (Figure 4C) which was associated with higher AMPD activity (Fig. 4D, left). These studies show that AMPK activity also regulates AMPD activity.

Of interest, ACC phosphorylation was not totally suppressed in AMPK-deficient cells indicating that other kinases may regulate ACC. This is consistent with previous reports that casein kinases and PKA phosphorylate (and inhibit) ACC [12], [26]. Another finding was that metformin reduced AMPD activity in the AMPK-deficient cells (Fig. 4D, right). This suggests that metformin may have a direct effect on AMPD independent of AMPK, consistent with a recent report [27]. Nevertheless, metformin was relatively ineffective at suppressing AMPD activity in AMPD-overexpressing cells, as noted in Fig. 3D.

### Mechanism of Fructose-Induced Fat Accumulation in HepG2 Cells

**Figure 5:**
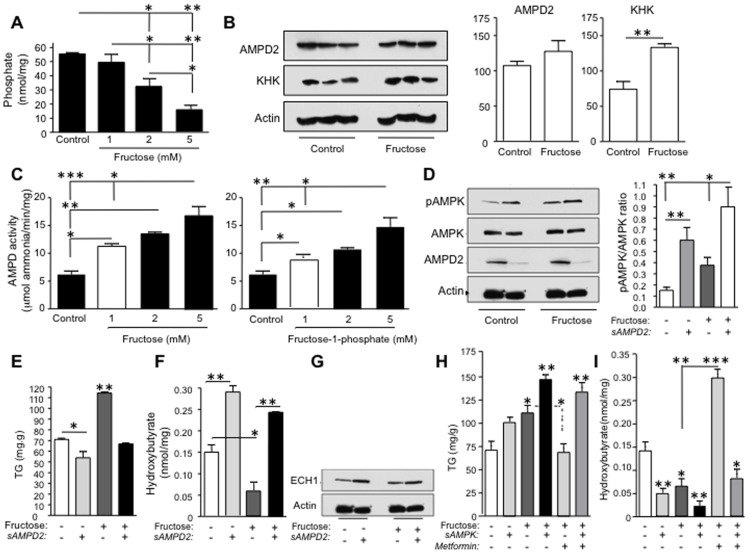
Fructose stimulates fat accumulation in HepG2 cells by activating AMPD2 and blocking AMPK and fat oxidation. A) Fructose reduces intracellular phosphate levels –a natural inhibitor of AMPD activity- in a dose-dependent manner. *p<0.05, **p<0.01. B) AMPD2 expression is not modified by fructose in human hepatocytes. As control, fructokinase (KHK) expression is significantly up-regulated. **p<0.01. C) Both fructose (1–5 mM) and fructose-1-phosphate (1–5 mM) -the product of fructokinase metabolism- significantly up-regulates the activity of AMPD2 in human hepatocytes. AMPD activity was measured after 30 minutes exposure to fructose in 50 µg protein lysates *p<0.05, **p<0.01. D) Fructose stimulates AMPK activity (as determined by phosphorylation at Thr172) that is further amplified in AMPD2 silenced cells. *p<0.05, **p<0.01. E) Fructose induces triglyceride accumulation in HepG2 cells that is blocked in AMPD2 deficient cells *p<0.05, **p<0.01 F) Fructose decreases β- hydroxybutyrate levels in HepG2 cells that are blocked in AMPD2 deficient cells *p<0.05, **p<0.01. G) ECH1 expression is up-regulated in AMPD2 deficient cells exposed to fructose. H) Silencing AMPK expression increases triglyceride accumulation in non-exposed and fructose exposed HepG2 cells that is not blocked with 10 µM metformin *p<0.05, **p<0.01 I) Silencing AMPK expression decreases intracellular β- hydroxybutyrate levels in non-exposed and fructose exposed HepG2 cells that is not blocked with 10 µM metformin *p<0.05, **p<0.01, ***p<0.001.

Fructose is unique from glucose in its ability to activate AMPD [28]. When fructose is metabolized, there is unchecked phosphorylation of fructose to fructose-1-phosphate by fructokinase (KHK), which is associated with transient intracellular phosphate depletion and GTP consumption that activates AMPD [29], [30]. As shown in Fig. 5A, HepG2 cells incubated with fructose for 72 hours demonstrated a significant decrease in intracellular phosphate in a dose-dependent manner. However, although fructose (5 mM) exposure resulted in a significant up-regulation of KHK, the protein expression of AMPD2 was not significantly modified (Fig. 5B). Nevertheless, AMPD activity was increased by fructose in a stepwise manner, an observation consistent with the known ability of reduced phosphate levels to activate this enzyme (Fig. 5C, left). While the stimulation of AMPD activity has been attributed to the phosphate and GTP depletion that occurs with fructose incubation, we also found that fructose-1-phosphate, a product of fructokinase-mediated fructose metabolism, also stimulated AMPD activity (Fig. 5C, right). These data document the marked ability of fructose to stimulate AMPD activity in HepG2 cells.

Interestingly, fructose-treated HepG2 cells also showed an increased in AMPK activity (pAMPK) despite the increased AMPD activity, documenting that while the two enzymes regulate each other, it is possible to have both activated at the same time (Fig. 5D) [31], [32]. Evidence that the two enzymes still regulate each other under fructose-treated conditions was tested by examining the effects of fructose on AMPK in HepG2 cells that had been silenced for AMPD. In these cells pAMPK levels was higher in response to fructose than observed in control HepG2 cells, and was associated with lower triglyceride and higher β-hydroxybutyrate levels (Fig. 5E–F). These studies show that AMPD2 is limiting the activation of AMPK in response to fructose.

We also performed the converse study, by examining the effect of fructose in HepG2 cells silenced for AMPK. In these cells fructose resulted in higher intracellular triglyceride accumulation and lower β-hydroxybutyrate levels than control HepG2 cells (Fig. 5G–I and Figure S2). Whereas metformin could reduce triglycerides levels and increase β-hydroxybutryate in control cells treated with fructose, it was ineffective in fructose-treated cells that had been silenced for AMPK (Fig. 5G–I). In summary, fructose activates both AMPD2 and AMPK in the liver, but the overall effect is consistent with AMPD being dominant, leading to fat accumulation and a reduction in overall β-fatty acid oxidation.

### AMPD2 Activation Produces Uric Acid which Negatively Regulates AMPK Activity

**Figure 6:**
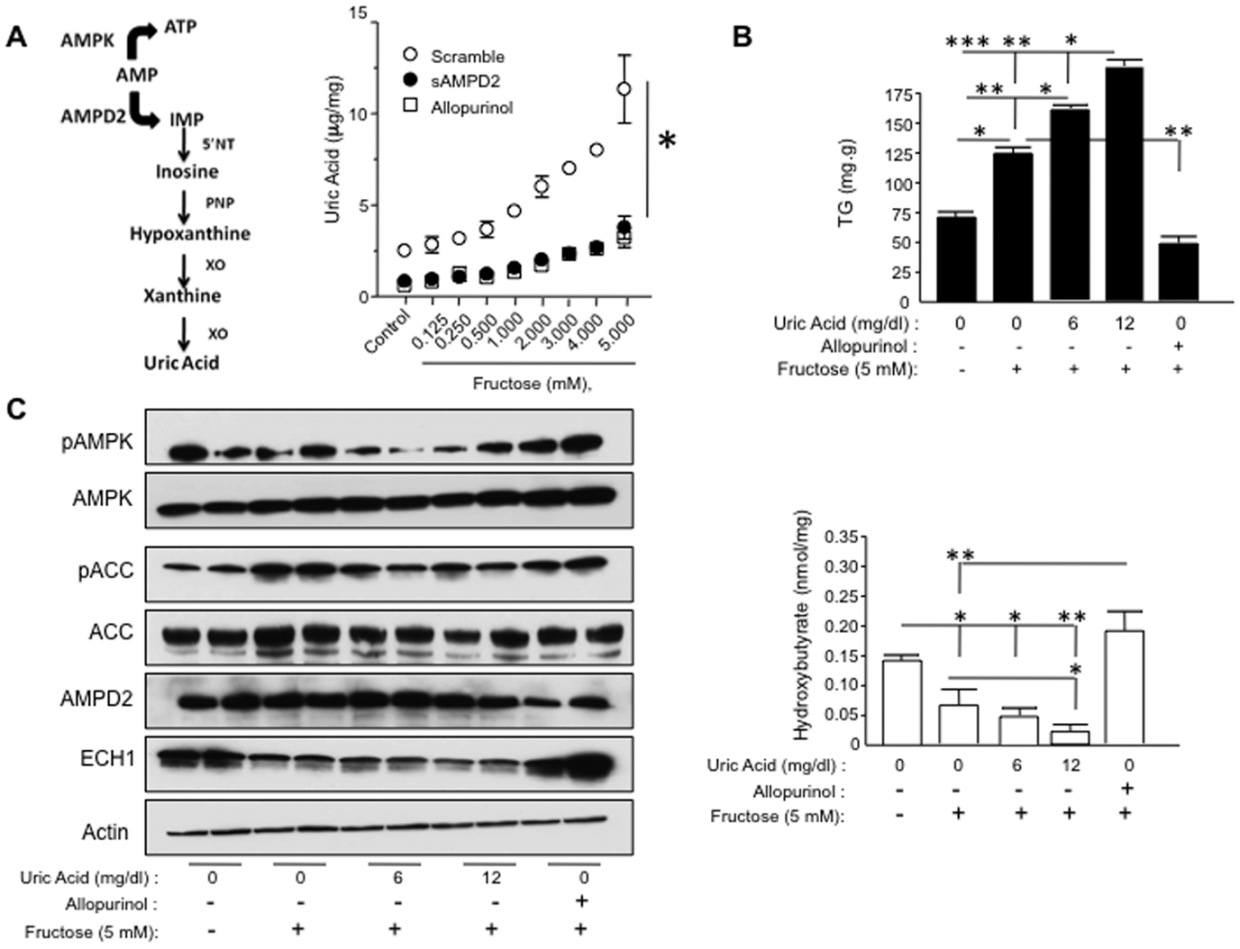
Uric acid negatively regulates AMPK activity. A) Schematic representation of downstream metabolites produced from AMP by AMPD2. Right, uric acid levels –the final product of this route- are increased in HepG2 cells exposed to fructose in a dose-dependent manner. Both inhibiting xanthine oxidase activity with allopurinol or silencing AMPD2 significantly inhibits uric acid generation. *p<0.05. B) Uric acid further increases fructose-induced triglyceride accumulation (top) and decreases β- hydroxybutyrate levels (bottom) in a dose-dependent manner while allopurinol blocks it. *p<0.05, **p<0.01, ***p<0.001 C) Representative western blot demonstrating that uric acid inhibits fructose-mediated activation of AMPK with significantly lower ACC phosphorylation at ser79 and ECH1 levels in a dose-dependent manner. In contrast, allopurinol (100 µM) further increases the activation of AMPK with significantly higher ACC phosphorylation at ser79 and ECH1 levels.

The stimulation of AMPD2 activity results in the conversion of AMP to inosine monophosphate (IMP), which is further degraded to uric acid (Fig. 6A, left). Fructose-treated HepG2 cells also showed an increase in intracellular uric acid that was prevented in AMPD2-deficient cells as well as in cells in which xanthine oxidoreductase (XOR) activity had been blocked with allopurinol (100 µM) (Fig. 6A, right). Since hyperuricemia is known to predict the development of fatty liver [33], [34], [35], we determined whether uric acid generated by AMPD activation could play a role on fatty liver generation by affecting AMPK and fat oxidation. Therefore, we exposed HepG2 cells to fructose alone (5 mM) or in combination with increasing levels of uric acid (0, 6 and 12 mg/dl) or allopurinol (100 µM) for 72 hours and determined AMPK activity, fat oxidation rates and intracellular triglyceride accumulation. As shown in Fig. 6B, the addition of uric acid to fructose enhanced triglyceride accumulation in a dose-dependent manner, and further lowered pACC, ECH1, pAMPK, and β-hydroxybutyrate levels (Fig. 6B–C), Conversely, allopurinol (100 µM) prevented fructose-induced triglyceride accumulation in HepG2 cells. These data show that uric acid regulates AMPK activity and fat oxidation.

**Figure 7:**
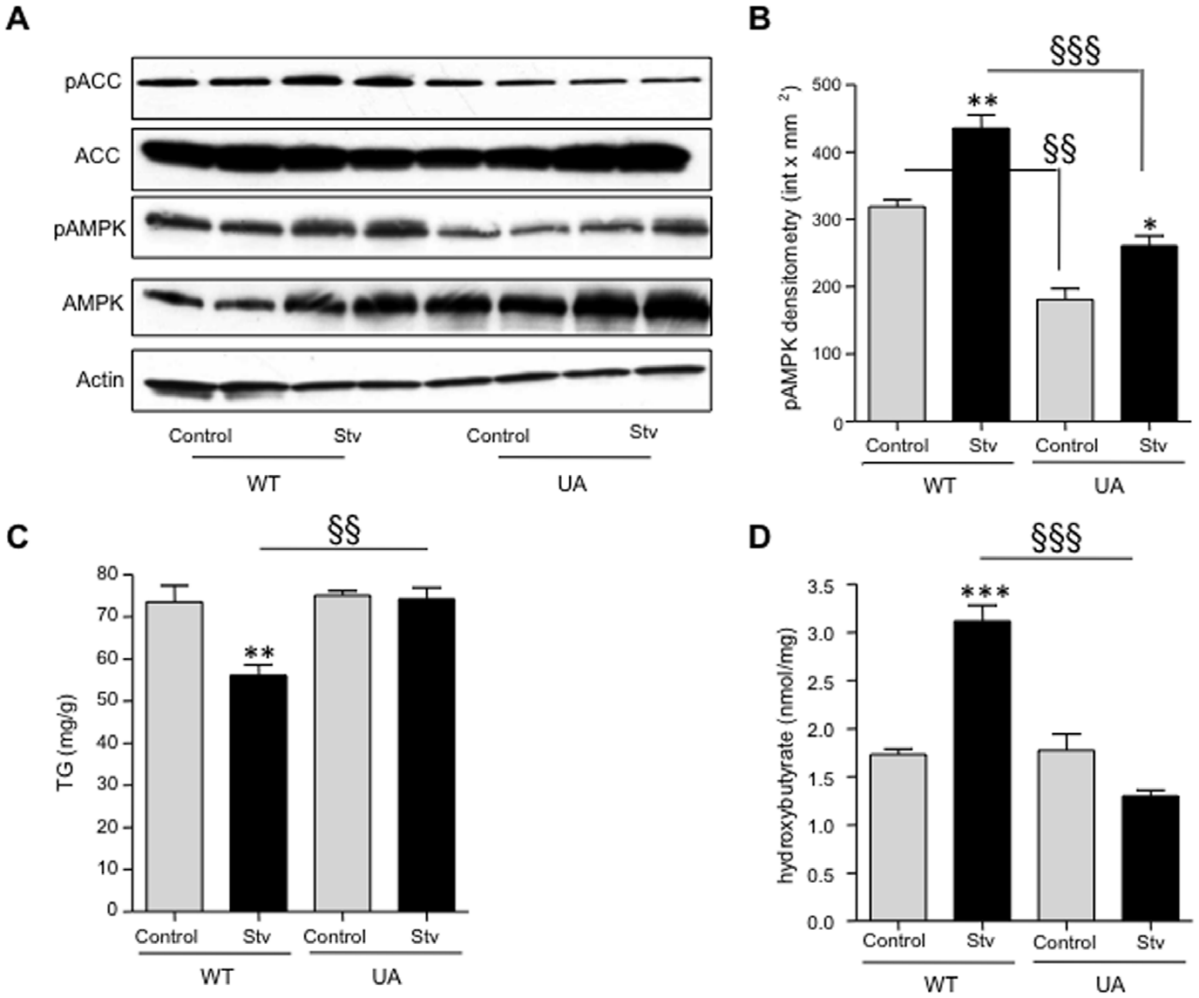
Uric acid down-regulates starvation-induced AMPK activation. HepG2 cells were exposed to 750 µmol/L uric acid for 48 hours prior 3 hour starvation (stv) and AMPK phosphorylation, intracellular TG and intracellular β-hydroxybutyrate levels were analyzed after starvation time. A–B) representative western blot and densitometry of phosphorylated AMPK in control and uric acid exposed cells undergoing starvation. *p<0.05, **p<0.01 versus control, §§ p<0.01 control vs control, §§§p<0.001 stv vs stv C) Intracellular TG levels in control and uric acid exposed cells undergoing starvation **p<0.01 versus control, §§p<0.01 stv vs stv. D) Intracellular β-hydroxybutyrate levels in control and uric acid exposed cells undergoing starvation ***p<0.001 versus control, §§§ p<0.001 stv vs stv.

After showing that uric acid negatively regulates AMPK activity in fructose-exposed HepG2 cells, we studied the potential role of uric acid in AMPK in settings different from fructose. To do that, we added uric acid to HepG2 cells and induced starvation –a well known activator of AMPK- for 3 hours by changing their regular growth medium to a fetal calf serum-free and glucose-free medium. As shown in Fig. 7, AMPK phosphorylation and phosphorylated ACC levels were significantly increased in non-uric acid exposed starving cells compared to cells maintained in regular growth medium. In contrast, no significant AMPK and ACC phosphorylation were observed in starving cells that were pre-exposed to uric acid indicating that uric acid may be a natural inhibitor of AMPK during starvation. Consistent with lower AMPK phosphorylation, starving cells exposed to uric acid failed to oxidize fat as determined by decreased β-hydroxybutyrate levels (Fig. 7D) and unchanged intracellular TG levels (Fig. 7C).

### Metformin Inhibits AMPD2 Activity in vivo and Reduces Fatty Liver in Sucrose-fed Rats

**Figure 8:**
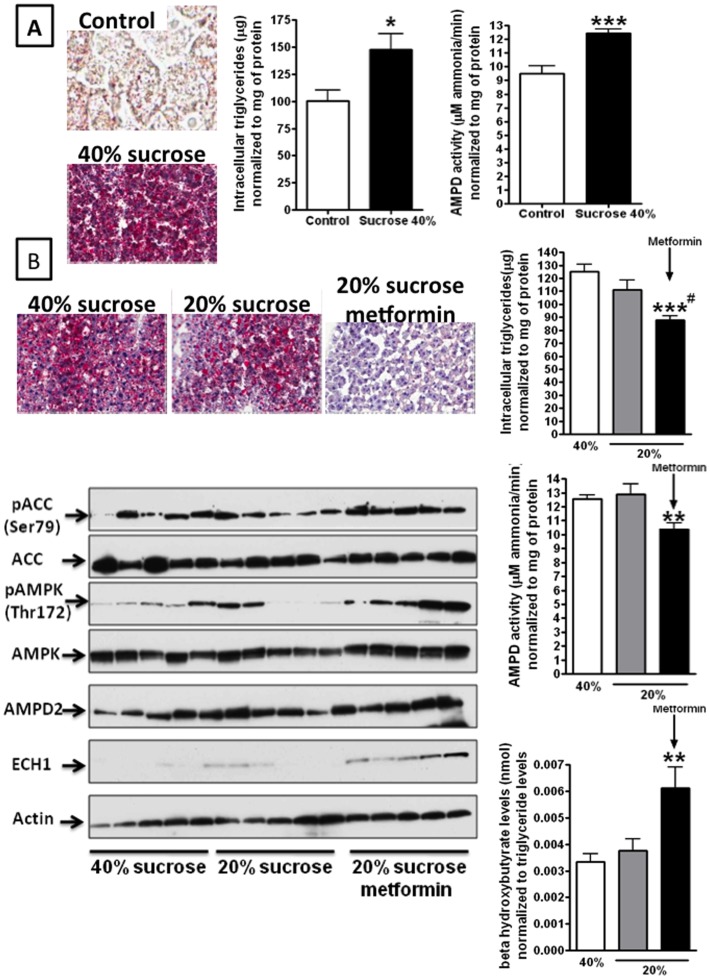
Metformin inhibits AMPD2 activity in vivo and reduces fatty liver in sucrose-fed rats. A–C) Rats fed with a 40% sucrose diet for 10 weeks develop fatty liver as determined by oil red-O staining and hepatic triglyceride determination. Activity of AMPD2 is significantly up-regulated in the livers of sucrose fed rats as compared to control rats. *p<0.05 versus control, *p<0.001 versus control. D–G) After 10 weeks, rats were maintained for more 4 weeks with a 40% sucrose diet or reduced to 20% alone or in combination with metformin (30 mg/kg). Reducing dietary sucrose to 20% does not significantly reduces fatty liver, AMPD2 activity or hepatic β- hydroxybutyrate levels. In contrast, metformin significantly reduces AMPD2 activity in the liver which is paralleled with reduction in liver fat accumulation and increase in production of β- hydroxybutyrate levels. H) Representative western blot depicting that treatment with metformin results in phosphorylation of AMPK at thr172, and with up-regulation of ECH1 levels **p<0.01 versus sucrose 40% and 20%, ***p<0.001 versus sucrose 40% and 20%, #p<0.01 versus sucrose 20%.

To characterize the role of AMPD2 in fatty liver *in vivo*, we fed rats a 40% sucrose diet (containing 20% glucose and 20% fructose) or 40% starch diet for 10 weeks. We selected this diet as compared to a pure fructose diet as the primary source of fructose in the American diet consists of sucrose or high fructose corn syrup, both of which consist of a combination of fructose and glucose. A subset of rats were sacrificed at 10 weeks and documented the presence of fatty liver in the sugar-fed rats as opposed to the starch-fed rats, as noted by Oil Red O staining (Fig. 8A) and intrahepatic triglyceride content (Fig. 8B). Hepatic AMPD activity was significantly higher in livers of sucrose-fed rats (Fig. 8C).

To determine whether the balance of AMPD2 and AMPK can be altered in this model to block the development of fatty liver, surviving rats were randomized to receive metformin (350 mg/kg/d) or vehicle for 4 additional weeks. We also reduced the sucrose intake to 20% in the diet in both groups during this period of time, as some dietary reduction in sugar would also be part of any treatment regimen given the strong association of fructose with fatty liver. A third group continued the 40% sucrose diet. As shown in Fig. 8D, the reduction in sucrose to 20% diet showed no or minimal effect on fatty liver compared to animals that continued to receive a diet of 40% sucrose, whereas the addition of metformin resulted in less fatty liver as determine by Oil Red O stain and intrahepatic triglyceride accumulation (Fig. 8E). Consistent with a reduction in intrahepatic fat levels, AMPD activity was significantly reduced in metformin-treated rats (Fig. 8F), and liver fat oxidation was significantly higher, as noted by increased P-AMPK and higher hepatic β-hydroxybutyrate levels (Fig. 8G–H).

## Discussion

In this study, we investigated the relationship of two AMP-dependent enzymes, AMPD2 and AMPK, in the development of fatty liver induced by fructose. Metabolic syndrome and fatty liver are associated with low hepatic ATP levels and increased nucleotide turnover resulting in substantial AMP generation [36], [37]. Stimulation of AMPK with agonists (AICAR, metformin) can prevent fatty liver in animal models by both enhancing fatty acid oxidation and inhibiting de novo lipogenesis [8], [38]. While it is well-known that AMPK stimulates fat oxidation and inhibits lipogenesis, no studies to date have investigated the interaction of AMPD2 and AMPK in fructose-induced fatty liver. In this paper, we demonstrate for the first time that AMPD2 has countering effects on fat metabolism in the liver. Specifically, we show that AMPD and AMPK counter-regulate each other, and that one of the mechanisms by which AMPD activation inhibits AMPK activity is by AMPD-dependent generation of uric acid. These studies provide potential insights into how fructose may induce fatty liver.

We first evaluated the effects of activation of AMPK and AMPD on fat oxidation in cultured hepatocytes (HepG2 cells). Activation of AMPK was shown by measuring the phosphorylation of threonine 172, which is widely regarded as a measurement for AMPK activation, and the downstream effects were determined by measuring protein levels of ECH-1 (an enzyme in β-fatty acid oxidation) and β-hydroxybutyrate as a direct measurement of fatty acid oxidation. Stimulation of AMPK using metformin could increase the phosphorylation of AMPK and increase fat oxidation rates –as determined by measuring intracellular β-hydroxybutyrate levels- by a mechanism that involved activation of PPARα, and this was not observed in AMPK deficient cells (Figs. 1 and 2). Of interest, we found that even though GW6471 could prevent ECH1 up-regulation induced by AMPK activation, AMPK phosphorylation was not inhibited by GW6471 (data not shown) indicating that AMPK acts primarily upstream of the transcription factor PPARα. In contrast, overexpression of AMPD2 in HepG2 cells resulted in increased AMPD activity and a reduction in fatty acid oxidation (Fig. 3). These studies showed that the activation of AMPK and AMPD result in opposing effects on fatty acid oxidation in HepG2 cells.

We next determined if AMPK and AMPD regulate each other. Silencing AMPD was found to increase AMPK activity in HepG2 cells (Fig. 4A). Likewise, silencing AMPK also increased AMPD activity (Fig. 4B). Thus, these two enzymes counter-regulate each other.

We also evaluated the effects of metformin in our system. Metformin is known to activate AMPK, possibly via stimulation of LKB1 [39]. We did document that metformin increased AMPK phosphorylation resulting in increased fat oxidation that was not observed in AMPK deficient cells. These studies confirm that metformin activates AMPK. Since AMPK was found to regulate AMPD, it is also not surprising that we found that metformin could reduce AMPD activity under various conditions, although metformin was less effective in AMPD-overexpressing cells. However, our observation that metformin could also reduce AMPD activity in AMPK deficient cells shows that metformin likely has direct effects on AMPD as well. Others have also recently reported that metformin may directly inhibit AMPD [27].

We next examined the relationship of AMPD and AMPK in fructose-mediated fat accumulation in HepG2 cells. Fructose is known to both increase AMPD activity as a consequence of ATP and GTP consumption with a reduction in intracellular phosphate levels [29], [30], and in this study we also show that AMPD activity is stimulated by fructose-1-phosphate, a product of fructose metabolism. In addition, AMPK is known to be activated during fructose metabolism due to the depletion of ATP, and consistent with this finding, we found that fructose stimulated P-AMPK. Evidence that the two enzyme pathways were regulating each other was shown, as the silencing of AMPD resulted in greater AMPK activation with increased fatty acid oxidation, whereas silencing of AMPK resulted in enhanced AMPD activity and less fatty acid oxidation (Fig. 5). In unmanipulated cells, the AMPD effect predominated, as evidence by intracellular triglyceride accumulation and a reduction in β-fatty acid oxidation. This is consistent with the known lipogenic effects of fructose [40].

One mechanism by which AMPD activation might inhibit AMPK would simply be by removing the availability of the AMP substrate. However, it is also possible that an AMPD product might act as an inhibitor of AMPK activity. One of the downstream products of AMPD is uric acid, which is known to predict both fatty liver and metabolic syndrome [33], [34], [35], [41]. We therefore examined the effect of uric acid on AMPK activity in HepG2 cells. We were able to show that the addition of uric acid to fructose resulted in a reduction in activated P-AMPK and less fatty acid oxidation, and conversely that lowering uric acid in fructose-treated cells had opposing effects (Fig. 6). These studies suggest intracellular uric acid is an endogenous inhibitor of AMPK. In this regard, we have confirmed that uric acid is able to inhibit AMPK phosphorylation in settings other than fructose exposure. As shown in Figure 7, we found that as compared to non-exposed cells, uric acid could block AMPK activation with a parallel decrease in the phosphorylation of ACC as well as fat oxidation in starving cells.

Finally, we examined the effect of metformin in sucrose-induced fatty liver in rats. Metformin was able to reduce the severity of fatty liver in association with a reduction in hepatic AMPD activity and an increase in p-AMPK (Fig. 8). These studies suggest that the alteration of the AMPD-AMPK balance in the liver may result in beneficial effects in hepatic steatosis.

In conclusion, AMPD and AMPK represent two enzymes that appear to have opposing effects of fatty acid oxidation, with AMPD inhibiting and AMPK increasing fatty acid oxidation. These two enzymes regulate each other. One AMPD product, uric acid, appears to be an endogenous inhibitor of AMPK. Fructose-induced fatty liver likely involves a state in which AMPD activity outweighs activation of AMPK. These studies provide new insights into the pathogenesis of hepatic steatosis.

## Methods

### Ethics Statement

All Animal experiments were performed according to protocols approved by the University of Colorado Animal Care and Use Committee.

### Materials

Cell culture medium (RPMI-1640), fetal calf serum, and antibiotics were from Gibco (Rockville, MD). Rabbit polyclonal antibody to AMPD2 (H00000271) was purchased from Novus (Littleton, CO), antibodies to KHK (HPA00740) and AMPD3 (SAB1300935) were purchased from Sigma (St Louis, MO), antibodies to lamin-A (SC-6214) and AMPD1 (SC-134225) were from Santa Cruz Biotechnology Inc. (Santa Cruz, CA), antibodies to β-actin (3700), total ACC (3676), phosphorylated ACC (Ser79, 3661), total AMPK (2532) and phosphorylated AMPK (Thr172, 2535) were from Cell Signaling (Danvers, MA) while the antibody to PPARα (3890) was from Millipore (Billerica, MA) and the one for ECH1 (11305-1-AP) was purchased from Proteintech. Secondary antibodies conjugated with horseradish peroxidase were from Cell Signaling. Fructose, fructose-1-phosphate, oleate, metfomin, GW6471, uric acid and allopurinol were purchased from Sigma. Uric acid (U0881, lot STBB4127) was confirmed negative for endotoxins (<0.05 eu/ml) and was dissolved and filter-sterilized in warm medium (37°C). Absence of uric acid crystals in the medium was confirmed by microscopy.

### Cell Culture, Silencing and Gene Overexpression in HepG2 Cells

The established human hepatocarcinoma cell line HepG2 was purchased from the American Type Culture Collection (ATCC, Manassas, VA). The cells were cultured in RPMI medium (17850 GIBCO, Grand Island, NY) supplemented with 10% fetal bovine serum (GIBCO) and 100 U/ml penicillin and 100 µg/ml streptomycin (GIBCO) in a humidified incubator with 5% CO_2_ at 37°C.

Prior to the experiments, cell viability was assessed by trypan blue treatment and counted using an automated cell counter to determine the toxicity of the compounds employed. No significant difference in cell viability was observed in HepG2 cells exposed to any of these compounds (data not shown). Expression of KHK, AMPKα1α2 and AMPD2 in HepG2 cells was stably silenced. Briefly, lentiviral particles codifying for a silencing sequence were obtained from Open Biosystems (KHK and AMPD2, Hunsville, AL) and Santa Cruz (AMPKα1α2). In all cases, HepG2 cells previously treated with polybrene (10 µg/ml) were exposed to the lentiviral particles for 24 hours for transduction. After exposure, medium was removed and cells were incubated in normal media in the presence of puromycin (2 µg/ml) to select transducted cells. Clones with greater than 90% silencing as assessed by western blot analysis were selected from colonies growing in plates from a 10-fold dilution series in media prepared with 2 µg/ml puromycin antibiotic. To overexpress ECH1 and AMPD2 in HepG2 cells, the coding sequence was inserted into the pLVXIRES puro vector by direct cloning between the EcoRI and NotI sites of the multicloning site. Correct cloning was confirmed by sequencing analysis. Seven micrograms of vector at 1 µg/µl was combined with the Lenti-X HTX packaging system (clontech) and HEK-293T cells maintained in tetracycline free medium were transfected with the components. Medium was collected at 48 hours and efficient production of lentiviral particles was assessed with the Lenti-X Gostix (clontech). As with the silencers, HepG2 cells were exposed to the virus and stable clones generated with puromycin. Controls refer to puromycin selected clones transducted with lentiviral particles containing scramble shRNA (for silencer control) or empty pLVXIRES Puro vector (for overexpression control). In experiments involving allopurinol, metformin, compound C and GW6471 cells were preincubated with these compounds for 8 hours prior exposure to fructose or uric acid.

### Protein Extraction and Western Blotting

Protein lysates were prepared from confluent cell cultures and rat livers employing MAP Kinase lysis buffer as previously described [42]. Sample protein content was determined by the BCA protein assay (Pierce). 40 µg of total protein was loaded per lane for SDS-PAGE (10% w/v) analysis and then transferred to PVDF membranes. Membranes were incubated with primary antibodies and visualized using a horseradish peroxidase secondary antibody and the HRP Immunstar® detection kit (Bio-Rad, Hercules, CA). Chemiluminescence was recorded with an Image Station 440CF and results analyzed with the 1D Image Software (Kodak Digital Science, Rochester, NY).

### Determination of Intracellular and Intrahepatic Uric Acid, β-hydroxybutyrate, Phosphate and Triglycerides

Cell lysates obtained with MAP kinase lysis buffer as well as serum collected from 8-hour fasting rats were analyzed using a VetAce autoanalyzer (Alfa Wassermann, West Caldwell, NJ) as previously described [43]. For triglyceride determination in liver and HepG2 cells, fat was solubilized by homogenization in 1 ml solution containing 5% nonidet P40 (NP-40) in water, slowly samples were exposed to 80–100°C in a water bath for 2–5 minutes until the NP-40 became cloudy, then cooled down to room temperature. Samples were then centrifuged for 2 minutes to remove any insoluble material. Triglyceride determination with the VetAce autoanalyzer consisted in their initial breakdown into fatty acids and glycerol. Glycerol is then oxidized to generate a product which reacts with the probe to generate color at 570 nm. Similarly, uric acid determination is based in the conversion of uric acid to allantoin, hydrogen peroxide (H_2_O_2_) and carbon dioxide by uricase. The H_2_O_2_ then, is determined by its reaction with the probe to generate color at approximately 571 nm. Values obtained were normalized per mg of soluble protein in the lysates. Hepatic β-hydrocybutyrate and phosphate levels in MAPK lysates was determined by enzymatical kits (K632 and K410, biovision).

### Determination of AMPD2 Activity

AMP deaminase activity was determined by estimating the production of ammonia by a modification of the method described by Chaney and Marbach [44] from cells collected in a buffer containing 150 mMKCl, 20 mM Tris-HCl, ImM EDTA, and 1 mM dithiothreitol. Briefly, the reaction mixture consisted of 25 mM sodium citrate, pH 6.0, 50 mM potassium chloride, and different concentration of AMP. The enzyme reaction was initiated by the addition of the enzyme solution and incubated at 37°C for 15 min. For determination of AMPD activation by fructose and fructose-1-phosphate, these compounds (1–5 mM) were added to the lysates 30 minutes before the assay for preincubation. The reaction was stopped with the addition of the phenol/hypochlorite reagents: Reagent A (100 mM phenol and 0.050 g/L sodium nitroprusside in H_2_O) was added, followed by reagent B (125 mM sodium hydroxide, 200 mM dibasicsodium phosphate, and 0.1% sodium hypochlorite in H_2_O) and incubated for 30 min at 25°C. The absorbance of the samples was measured at 625 nm with a spectrophotometer. To determine the absolute specific activity of ammonia production (micromoles ammonia/min), a calibration curve was determined in the range of 5 µM to 1 mM of ammonia.

Data obtained from this assay was confirmed by HPLC by monitoring the enzyme-mediated formation of IMP as described [45]. Briefly, the assay was performed at 37°C for 15 minutes in a buffer contained 27 mM imidazole-HCL (pH 6.5), 100 mM KCL, 1 mM DTT, 5 µM EHNA, and 12.5 mM AMP. The reaction was terminated by the addition of 2N perchloric acid, the acid extract was neutralized with a mixture of tri-n-octylamine: 1,1,2-trichlorotrifluoroethene (55∶ 145 v/v), and IMP was separated using HPLC. The column used was a ZORBAX Eclipse XDB-C18 with a mean particular size of 5 µm (Agilent Technologies, Santa Clara, CA). The effluent was monitored at 254 nm; chromatographic peaks were identified according to retention times of standards containing known amounts of AMP and IMP.

### Rat Experiments

400 g male Sprague-Dawley breeder rats (Charles Rivers, Wilmington, MA) were housed in the animal facility at the University of Colorado. Rats were kept under temperature- and humidity-controlled specific pathogen-free conditions and maintained on a 12 hour light-dark cycle. Animals received normal chow containing 18% protein and 6% fat (3.1 kcal/g of metabolizable energy) (2918, Harlan Laboratories, Madison, WI). All experimental protocols were approved by the University of Colorado Animal Care and Use Committee. Rats were randomly divided into two main groups: Control (*n* = 6), sucrose 40% in chow (n = 24) and pair-fed equal amounts throughout the study. Ten weeks after starting the experiment control rats and 6 sucrose rats were sacrificed to confirm development of fatty liver. Some data on this set of animals has already been reported [46]. The remaining 18 rats were subdivided into three groups, sucrose 40% (n = 6), sucrose 20% (n = 6) and sucrose 20% plus metformin (350 mg/kg, n = 6). Water and food consumption was closely monitorized. At sacrifice, serum and livers were collected. Liver tissue was immediately processed for oil RedO staining and snap frozen for protein, AMPD activity, β-hydroxyutyrate and triglyceride determination.

### Liver Oil Red o Staining

Liver tissue collected under isoflurane anesthesia was embedded in Optimal Cutting Temperature gel (OCT; Sakura Finetek, Torrance, CA) and frozen in liquid nitrogen. Air-dried cryostat tissue sections (8 *µ*m) were dipped in formalin, washed with running tap water, rinsed with 60% isopropanol, and stained with oil red O and counterstained with hematoxylin. Macrovesicular fat deposition was defined as the presence of lipid vacuoles that are larger than the nucleus and usually displaces it to the periphery of the cell.

### Immunofluorescence of ECH1-GFP and Confocal Microscopy for Neutral Lipid Determination

Human ECH1 coding sequence was was inserted into the pAcGFPN1 (clontech) vector by direct cloning between the XhoI and the EcoRI sites of the multicloning site. Correct cloning was confirmed by sequencing analysis. HepG2 cells were grown in coverslips to 60% confluency and transfected with lipofectamine 200 as per manufacturer’s protocol (Invitrogen) obtaining 10–15% transfection efficiency. After exposure of cells to oleate, cells were stained with the fluorescent lipid marker lipidtox red (molecular probes) as per manufacturer’s protocol. Cells were imaged with a confocal microscope for colocalization studies. Imaging and analysis was performed with a laser-scanning confocal microscope (LSM510, Carl Zeiss, Thornwood, NY) with a 40x water immersion objective and the corresponding postacquisition software.

### Statistics and Data Analysis

All data are presented as the mean±standard error of the mean (SEM). Data graphics and statistical analysis were performed using Instat (version 3.0) and Prism 5 (both Graph Pad Software, San Diego, CA). Data was analyzed for normality tests and using the Tukey-Kramer multiple comparison test. Multiple group corrections were performed using the method of Bartlett. In most cases experiments were performed 3 times with independent replicates. Total data points (n) are identified in figure legends. All experiments with cultured cells were performed at least twice and in triplicates (n>6).

## Supporting Information

Figure S1 **Metformin increases ECH1 expression in human hepatocytes through AMPK activation.** A) Representative quantitaion of pACC/ACC and pAMPK/APK ratio in HepG2 cells exposed to oleate and/or metformin B–C) Representative western blot and densitometry of total and phosphorylated AMPK, phosphorylated ACC and ECH1 in control and AMPK deficient cells (sAMPK) in the presence of metformin (10 µM) and/or oleate (250 µM). *p<0.05, *p<0.01, a p<0.001 versus control cells.
(TIF)

Figure S2 **Fructose reduces intracellular β-hydroxybutyrate levels in an AMPK-dependent pathway.** A) Intracellular β-hydroxybutyrate levels in control and AMPK deficient cells at basal conditions or fructose-exposed (72 hr) conditions. *p<0.05 versus white columns. #p<0.05.
(TIF)

## References

[1] Ford ES, Giles WH, Mokdad AH (2004) Increasing prevalence of the metabolic syndrome among u.s. Adults. Diabetes Care 27: 2444–2449. 15451914 10.2337/diacare.27.10.2444

[2] Polyzos SA, Kountouras J, Deretzi G, Zavos C, Mantzoros CS (2012) The Emerging Role of Endocrine Disruptors in Pathogenesis of Insulin Resistance: A Concept Implicating Nonalcoholic Fatty Liver Disease. Curr Mol Med. 10.2174/156652412798376161 22082482

[3] Caballeria L, Auladell MA, Toran P, Pera G, Miranda D, et al (2008) Risk factors associated with non-alcoholic fatty liver disease in subjects from primary care units. A case-control study. BMC Gastroenterol 8: 44. 18831772 10.1186/1471-230X-8-44 PMC2569953

[4] Ix JH, Sharma K (2010) Mechanisms linking obesity, chronic kidney disease, and fatty liver disease: the roles of fetuin-A, adiponectin, and AMPK. J Am Soc Nephrol 21: 406–412. 20150538 10.1681/ASN.2009080820 PMC4473254

[5] Musso G, Gambino R, Cassader M (2010) Emerging molecular targets for the treatment of nonalcoholic fatty liver disease. Annu Rev Med 61: 375–392. 20059344 10.1146/annurev.med.60.101107.134820

[6] Viollet B, Foretz M, Guigas B, Horman S, Dentin R, et al (2006) Activation of AMP-activated protein kinase in the liver: a new strategy for the management of metabolic hepatic disorders. J Physiol 574: 41–53. 16644802 10.1113/jphysiol.2006.108506 PMC1817784

[7] Hu M, Wang F, Li X, Rogers CQ, Liang X, et al. (2012) Regulation of hepatic lipin-1 by ethanol: Role of AMPK-SREBP-1 signaling. Hepatology. 10.1002/hep.24708 PMC3253249 21953514

[8] Li Y, Xu S, Mihaylova MM, Zheng B, Hou X, et al (2011) AMPK phosphorylates and inhibits SREBP activity to attenuate hepatic steatosis and atherosclerosis in diet-induced insulin-resistant mice. Cell Metab 13: 376–388. 21459323 10.1016/j.cmet.2011.03.009 PMC3086578

[9] Jung EJ, Kwon SW, Jung BH, Oh SH, Lee BH (2011) Role of the AMPK/SREBP-1 pathway in the development of orotic acid-induced fatty liver. J Lipid Res 52: 1617–1625. 21757781 10.1194/jlr.M015263 PMC3151682

[10] Kawaguchi T, Osatomi K, Yamashita H, Kabashima T, Uyeda K (2002) Mechanism for fatty acid “sparing” effect on glucose-induced transcription: regulation of carbohydrate-responsive element-binding protein by AMP-activated protein kinase. J Biol Chem 277: 3829–3835. 11724780 10.1074/jbc.M107895200

[11] Abu-Elheiga L, Matzuk MM, Abo-Hashema KA, Wakil SJ (2001) Continuous fatty acid oxidation and reduced fat storage in mice lacking acetyl-CoA carboxylase 2. Science 291: 2613–2616. 11283375 10.1126/science.1056843

[12] Ha J, Daniel S, Broyles SS, Kim KH (1994) Critical phosphorylation sites for acetyl-CoA carboxylase activity. J Biol Chem 269: 22162–22168. 7915280

[13] Barroso E, Rodriguez-Calvo R, Serrano-Marco L, Astudillo AM, Balsinde J, et al (2011) The PPARbeta/delta activator GW501516 prevents the down-regulation of AMPK caused by a high-fat diet in liver and amplifies the PGC-1alpha-Lipin 1-PPARalpha pathway leading to increased fatty acid oxidation. Endocrinology 152: 1848–1859. 21363937 10.1210/en.2010-1468

[14] Buler M, Aatsinki SM, Skoumal R, Komka Z, Toth M, et al. (2012) Energy sensing factors coactivator PGC-1alpha and AMP-activated protein kinase control expression of inflammatory mediators in liver: induction of Interleukin 1 receptor antagonist. J Biol Chem. 10.1074/jbc.M111.302356 PMC3265866 22117073

[15] Gross M (1994) Molecular biology of AMP deaminase deficiency. Pharm World Sci 16: 55–61. 8032342 10.1007/BF01880656

[16] Morisaki T, Sabina RL, Holmes EW (1990) Adenylate deaminase. A multigene family in humans and rats. J Biol Chem 265: 11482–11486. 2365682

[17] Nakagawa T, Hu H, Zharikov S, Tuttle KR, Short RA, et al (2006) A causal role for uric acid in fructose-induced metabolic syndrome. Am J Physiol Renal Physiol 290: F625–631. 16234313 10.1152/ajprenal.00140.2005

[18] Sanchez-Lozada LG, Mu W, Roncal C, Sautin YY, Abdelmalek M, et al (2010) Comparison of free fructose and glucose to sucrose in the ability to cause fatty liver. Eur J Nutr 49: 1–9. 19626358 10.1007/s00394-009-0042-x PMC2805058

[19] Benner SA, Ellington AD, Tauer A (1989) Modern metabolism as a palimpsest of the RNA world. Proc Natl Acad Sci U S A 86: 7054–7058. 2476811 10.1073/pnas.86.18.7054 PMC297992

[20] Zhou G, Myers R, Li Y, Chen Y, Shen X, et al (2001) Role of AMP-activated protein kinase in mechanism of metformin action. J Clin Invest 108: 1167–1174. 11602624 10.1172/JCI13505 PMC209533

[21] Hawley SA, Davison M, Woods A, Davies SP, Beri RK, et al (1996) Characterization of the AMP-activated protein kinase kinase from rat liver and identification of threonine 172 as the major site at which it phosphorylates AMP-activated protein kinase. J Biol Chem 271: 27879–27887. 8910387 10.1074/jbc.271.44.27879

[22] Filppula SA, Yagi AI, Kilpelainen SH, Novikov D, FitzPatrick DR, et al (1998) Delta3,5-delta2,4-dienoyl-CoA isomerase from rat liver. Molecular characterization. J Biol Chem 273: 349–355. 9417087 10.1074/jbc.273.1.349

[23] Krebs HA, Hems R (1970) Fatty acid metabolism in the perfused rat liver. Biochem J 119: 525–533. 5500312 10.1042/bj1190525 PMC1179383

[24] Jia Y, Qi C, Zhang Z, Hashimoto T, Rao MS, et al (2003) Overexpression of peroxisome proliferator-activated receptor-alpha (PPARalpha)-regulated genes in liver in the absence of peroxisome proliferation in mice deficient in both L- and D-forms of enoyl-CoA hydratase/dehydrogenase enzymes of peroxisomal beta-oxidation system. J Biol Chem 278: 47232–47239. 14500732 10.1074/jbc.M306363200

[25] Miyazaki M, Nakagawa I, Koga S, Kasahara Y, Patricelli MP (2010) Proteomics analysis of cardiac muscle from rats with peroxisomal proliferator-activated receptor alpha (PPARalpha) stimulation. J Toxicol Sci 35: 131–135. 20118634 10.2131/jts.35.131

[26] Tipper JP, Bacon GW, Witters LA (1983) Phosphorylation of acetyl-coenzyme A carboxylase by casein kinase I and casein kinase II. Arch Biochem Biophys 227: 386–396. 6141763 10.1016/0003-9861(83)90468-x

[27] Ouyang J, Parakhia RA, Ochs RS (2011) Metformin activates AMP kinase through inhibition of AMP deaminase. J Biol Chem 286: 1–11. 21059655 10.1074/jbc.M110.121806 PMC3012963

[28] Van den Berghe G (1986) Fructose: metabolism and short-term effects on carbohydrate and purine metabolic pathways. Prog Biochem Pharmacol 21: 1–32. 3523498

[29] van den Berghe G, Bronfman M, Vanneste R, Hers HG (1977) The mechanism of adenosine triphosphate depletion in the liver after a load of fructose. A kinetic study of liver adenylate deaminase. Biochem J 162: 601–609. 869906 10.1042/bj1620601 PMC1164643

[30] Phillips MI, Davies DR (1985) The mechanism of guanosine triphosphate depletion in the liver after a fructose load. The role of fructokinase. Biochem J 228: 667–671. 2992452 10.1042/bj2280667 PMC1145036

[31] Gillespie JG, Hardie DG (1992) Phosphorylation and inactivation of HMG-CoA reductase at the AMP-activated protein kinase site in response to fructose treatment of isolated rat hepatocytes. FEBS Lett 306: 59–62. 1628744 10.1016/0014-5793(92)80837-7

[32] Moore F, Weekes J, Hardie DG (1991) Evidence that AMP triggers phosphorylation as well as direct allosteric activation of rat liver AMP-activated protein kinase. A sensitive mechanism to protect the cell against ATP depletion. Eur J Biochem 199: 691–697. 1678349 10.1111/j.1432-1033.1991.tb16172.x

[33] Ouyang X, Cirillo P, Sautin Y, McCall S, Bruchette JL, et al (2008) Fructose consumption as a risk factor for non-alcoholic fatty liver disease. J Hepatol 48: 993–999. 18395287 10.1016/j.jhep.2008.02.011 PMC2423467

[34] Lonardo A, Loria P, Leonardi F, Borsatti A, Neri P, et al (2002) Fasting insulin and uric acid levels but not indices of iron metabolism are independent predictors of non-alcoholic fatty liver disease. A case-control study. Dig Liver Dis 34: 204–211. 11990393 10.1016/s1590-8658(02)80194-3

[35] Xu C, Yu C, Xu L, Miao M, Li Y (2010) High serum uric acid increases the risk for nonalcoholic Fatty liver disease: a prospective observational study. PLoS One 5: e11578. 20644649 10.1371/journal.pone.0011578 PMC2904389

[36] Schmid AI, Szendroedi J, Chmelik M, Krssak M, Moser E, et al (2011) Liver ATP synthesis is lower and relates to insulin sensitivity in patients with type 2 diabetes. Diabetes Care 34: 448–453. 21216854 10.2337/dc10-1076 PMC3024365

[37] Yang S, Lin H, Diehl AM (2001) Fatty liver vulnerability to endotoxin-induced damage despite NF-kappaB induction and inhibited caspase 3 activation. Am J Physiol Gastrointest Liver Physiol 281: G382–392. 11447019 10.1152/ajpgi.2001.281.2.G382

[38] Yu LF, Qiu BY, Nan FJ, Li J (2010) AMPK activators as novel therapeutics for type 2 diabetes. Curr Top Med Chem 10: 397–410. 20180761 10.2174/156802610790980611

[39] Shaw RJ, Lamia KA, Vasquez D, Koo SH, Bardeesy N, et al (2005) The kinase LKB1 mediates glucose homeostasis in liver and therapeutic effects of metformin. Science 310: 1642–1646. 16308421 10.1126/science.1120781 PMC3074427

[40] Stanhope KL, Havel PJ (2008) Fructose consumption: potential mechanisms for its effects to increase visceral adiposity and induce dyslipidemia and insulin resistance. Curr Opin Lipidol 19: 16–24. 18196982 10.1097/MOL.0b013e3282f2b24a PMC4151171

[41] Kodama S, Saito K, Yachi Y, Asumi M, Sugawara A, et al (2009) Association between serum uric acid and development of type 2 diabetes. Diabetes Care 32: 1737–1742. 19549729 10.2337/dc09-0288 PMC2732137

[42] Lanaspa MA, Andres-Hernando A, Rivard CJ, Dai Y, Li N, et al (2009) ZAC1 is up-regulated by hypertonicity and decreases sorbitol dehydrogenase expression, allowing accumulation of sorbitol in kidney cells. J Biol Chem 284: 19974–19981. 19423711 10.1074/jbc.M109.001792 PMC2740423

[43] Roncal-Jimenez CA, Lanaspa MA, Rivard CJ, Nakagawa T, Sanchez-Lozada LG, et al (2011) Sucrose induces fatty liver and pancreatic inflammation in male breeder rats independent of excess energy intake. Metabolism 60: 1259–1270. 21489572 10.1016/j.metabol.2011.01.008 PMC3137694

[44] Chaney AL, Marbach EP (1962) Modified reagents for determination of urea and ammonia. Clin Chem 8: 130–132. 13878063

[45] Raffin JP, Thebault MT (1991) A specific AMP deaminase assay and its application to tissue homogenates. Comp Biochem Physiol B 99: 125–127. 1959322 10.1016/0305-0491(91)90016-7

[46] Johnson RJ, Lanaspa MA, Roncal-Jimenez C, Sanchez-Lozada LG (2012) Effects of excessive fructose intake on health. Ann Intern Med 156: 905. 22711095 10.7326/0003-4819-156-12-201206190-00024

