# Supplementary figures and images for: Counteracting Roles of AMP Deaminase and AMP Kinase in the Development of Fatty Liver
Source: PLoS One. 2012 Nov 9;7(11):e48801. doi: 10.1371/journal.pone.0048801 (PMC3494720; doi:10.1371/journal.pone.0048801)

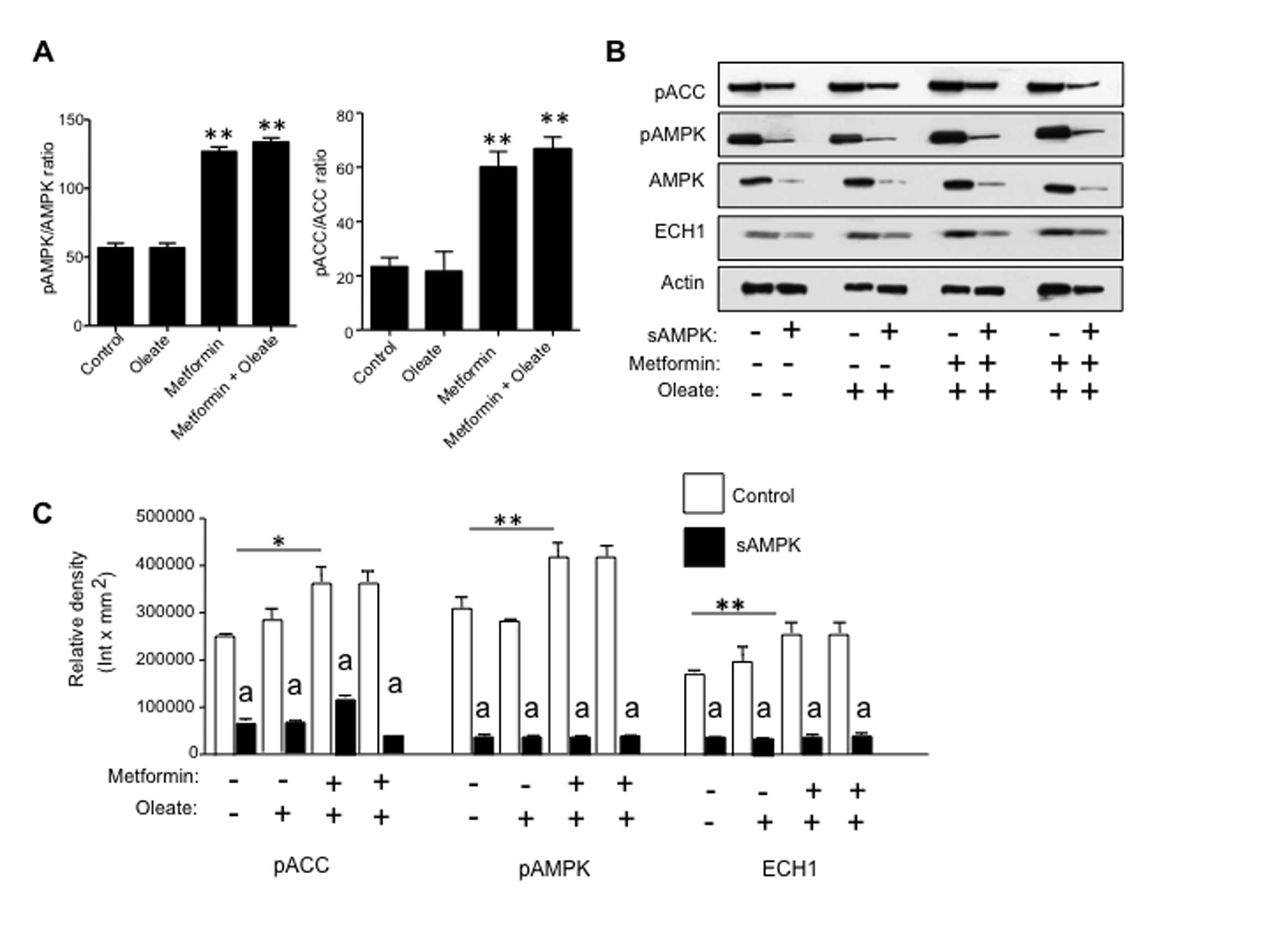

Supplement: Figure S1 — Metformin increases ECH1 expression in human hepatocytes through AMPK activation. A) Representative quantitaion of pACC/ACC and pAMPK/APK ratio in HepG2 cells exposed to oleate and/or metformin B–C) Representative western blot and densitometry of total and phosphorylated AMPK, phosphorylated ACC and ECH1 in control and AMPK deficient cells (sAMPK) in the presence of metformin (10 µM) and/or oleate (250 µM). *p<0.05, *p<0.01, a p<0.001 versus control cells. (TIF) [file pone.0048801.s001.tif]

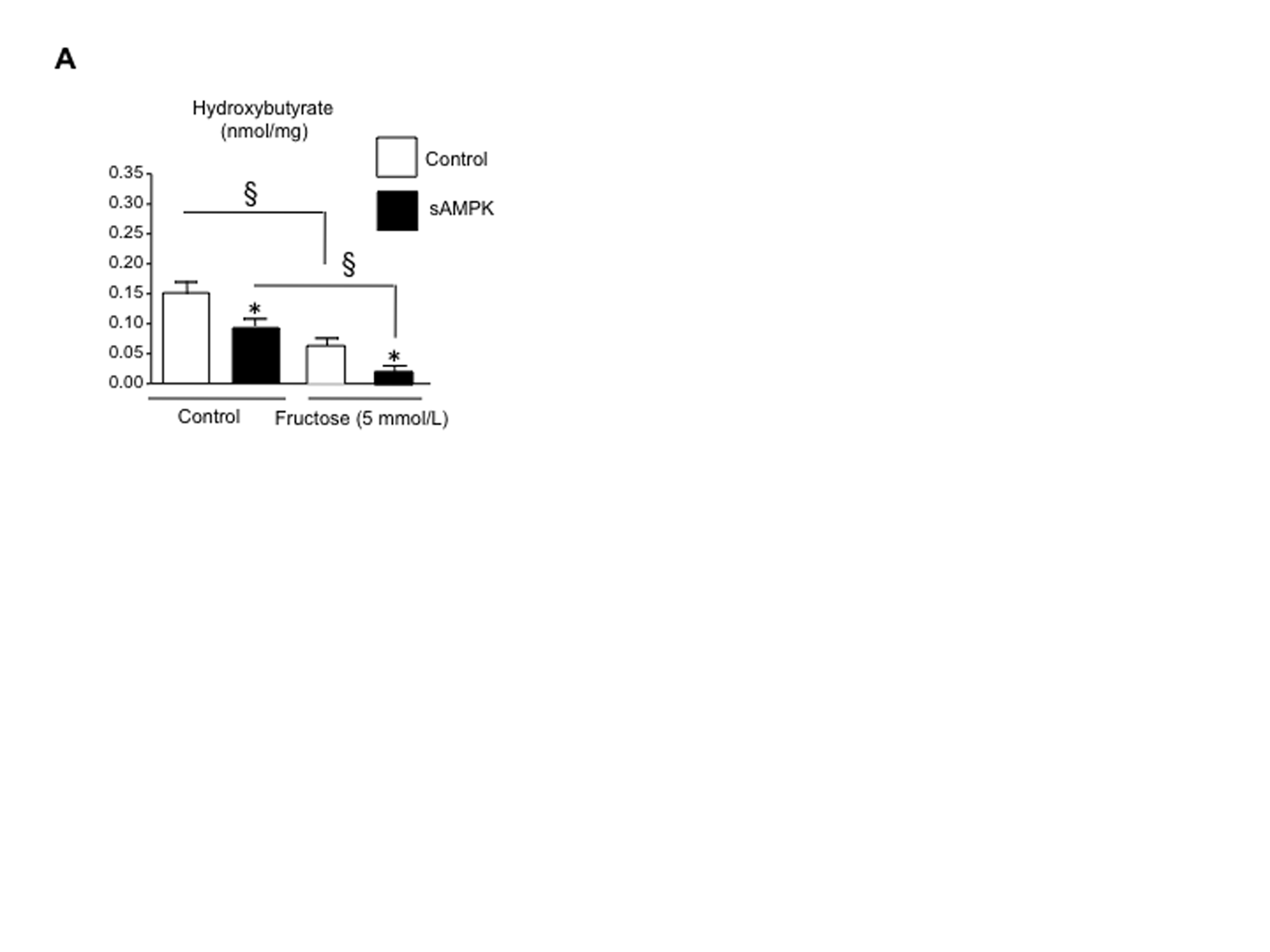

Supplement: Figure S2 — Fructose reduces intracellular β-hydroxybutyrate levels in an AMPK-dependent pathway. A) Intracellular β-hydroxybutyrate levels in control and AMPK deficient cells at basal conditions or fructose-exposed (72 hr) conditions. *p<0.05 versus white columns. #p<0.05. (TIF) [file pone.0048801.s002.tif]
